# Monoclonal outbreak caused by ceftazidime-avibactam resistant bla_OXA-48_-positive, carbapenem-resistant Klebsiella pneumoniae in an intensive care unit

**DOI:** 10.3205/dgkh000610

**Published:** 2026-01-09

**Authors:** Bahise Çağla Taşkın Dalgıç, Elif Seren Tanriverdi, İlayda Budak, Gülgün Yenişehirli, Barış Otlu

**Affiliations:** 1 Microbiology Laboratory Unit, Manisa Public Health Laboratory, Manisa, Turkey; 2 Department of Medical Microbiology, Inonu University, Faculty of Medicine, Malatya, Turkey; 3 Department of Medical Microbiology, Tokat Gaziosmanpasa University, Faculty of Medicine, Tokat, Turkey

**Keywords:** Carbapenem-resistant K. pneumoniae, ceftazidime-avibactam, hospital outbreak, intensive care unit, OXA-48

## Abstract

**Purpose:**

This study aimed to determine the ceftazidime-avibactam susceptibilities and clonal relationships of *bla_OXA-48_*-positive, carbapenem-resistant *K. pneumaniae* (CRKP) isolates.

**Methods:**

Fifteen *K. pneumaniae* isolates from various clinical samples, determined as *bla_OXA-48_*-positive CRKP by VITEK 2 compact system and multiplex PCR, were included in the study. Colistin susceptibility of isolates was tested using the broth microdilution method. The Kirby-Bauer disk diffusion test was performed for CAZ-AVI susceptibility. Arbitrary primer sequence-based PCR (AP-PCR) was used to investigate genotypic similarity between the isolates.

**Results:**

All 15 *bla_OXA-48_*-positive CRKP isolates were resistant to meropenem, ertapenem, imipenem, amikacin, gentamicin, ciprofloxacin and levoflokxacin. Four of the CRKP isolates were susceptible to SXT and colistin. 73.3% (11/15) of the CRKP isolates were found to be resistant to CAZ-AVI. One of the 15 *bla_OXA-48_*-positive CRKP isolates was also a *bla_NDM_* carrier. AP-PCR revealed that 15 isolates showed five different genotypes. Eleven of the isolates were classified under genotype 1, leading to a clustering rate of 73.3%. Isolates in the genotype 1 group were defined as outbreak isolates. All but one of the genotype 1 outbreak isolates were resistant to all antibiotics studied, including CAZ-AVI and colistin.

**Conclusion:**

This study evaluated an intensive care unit outbreak caused by a clone resistant to CAZ-AVI, which has been reported to have susceptibility rates of up to 100% in OXA-48-producing CRKP isolates and is suggested as first choice therapy. To prevent the spread of CAZ-AVI-resistant CRKP isolates, it is essential to conduct active surveillance studies in hospitals. Especially ICUs – where these strains are common – should be routinely screened. Infection control practices in general must also be tightened.

## Zusammenfassung

**Zielsetzung:**

Ziel der Studie war die Bestimmung der Ceftazidim-Avibactam-Empfindlichkeit und der klonalen Verwandtschaftsverhältnisse von *bla_OXA-48_*-positiven Carbapenem-resistenten *K. pneumoniae*-Isolaten (CRKP).

**Methode:**

Fünfzehn *K. pneumoniae* Isolate aus verschiedenen klinischen Proben, die mittels VITEK 2 Compact System und Multiplex-PCR als *bla_OXA-48_*-positive CRKP-Isolaten identifiziert wurden, wurden in die Studie einbezogen. Die Colistin-Empfindlichkeit der Isolate wurde mittels Bouillon-Mikrodilution untersucht. Die CAZ-AVI-Empfindlichkeit wurde mit dem Kirby-Bauer-Disk-Diffusionstest bestimmt. Die genotypische Ähnlichkeit zwischen den Isolaten wurde mittels Arbitrary-Primer-Sequenz-basierter PCR (AP-PCR) untersucht.

**Ergebnisse:**

Alle 15 *bla_OXA-48_*-positiven CRKP-Isolate waren resistent gegen Meropenem, Ertapenem, Imipenem, Amikacin, Gentamicin, Ciprofloxacin und Levofloxacin. Vier der CRKP-Isolate waren empfindlich gegenüber SXT und Colistin. 73,3% (11/15) der CRKP-Isolate waren resistent gegenüber CAZ-AVI. Eines der 15 *bla_OXA-48_*-positiven CRKP-Isolate war gleichzeitig *bla_NDM_*-Träger. Mittels AP-PCR zeigten 15 Isolate fünf verschiedene Genotypen. Elf der Isolate wurden dem Genotyp 1 zugeordnet, was zu einer Clusterrate von 73,3% führte. Isolate der Genotyp-1-Gruppe wurden als Ausbruchsisolate definiert. Alle Ausbruchsisolate des Genotyps 1, mit Ausnahme eines Isolats, waren gegen alle untersuchten Antibiotika einschließlich CAZ-AVI und Colistin resistent.

**Schlussfolgerung:**

Die Studie zeigt einen Ausbruch auf einer Intensivstation, verursacht durch einen Klon, der gegen CAZ-AVI resistent ist. CAZ-AVI weist in OXA-48-produzierenden CRKP-Isolaten Empfindlichkeitsraten von bis zu 100% auf; allerdings wird es bisher als Therapie der ersten Wahl empfohlen. Um die Ausbreitung CAZ-AVI-resistenter CRKP-Isolate zu verhindern, sind Surveillancesstudien in Krankenhäusern, insbesondere routinemäßige Screenings auf Intensivstationen, auf denen diese Stämme häufig vorkommen, sowie verstärkte Infektionskontrollmaßnahmen unerlässlich.

## Introduction

Globally, the spread of carbapenem-resistant *K. pneumoniae* (CRKP) is rapidly increasing, and carbapenemase types vary between countries and regions. Among the carbapenemase types in *K. pneumoniae* isolates, KPC continues to be an endemic species in many parts of the world, especially in the USA [1], [2]. In Turkey, unlike the rest of the world, OXA-48-producing* K. pneumoniae* strains have been dominant for many years [3], [4]. Investigation of carbapenemase production and determination of its type in a CRKP isolate are very important in deciding which drug to choose for treatment [5]. To solve the resistance problem in Gram negative pathogens, new beta-lactamase inhibitors have been combined with known cephalosporins. The resulting ceftazidime-avibactam (CAZ-AVI) has shown significant in-vitro activity on CRKP isolates due to its broad enzyme inhibition effect, for instance, on OXA-48, KPC, ESBL and Amp C, but not MBL [5], [6]. 

In the IDSA 2023 guideline for treatment of Gram-negative infections, ceftazidime-avibactam is recommended for use in the treatment of infections other than those of the urinary tract, due to carbapenem-resistant Enterobactericeae (CRE). The same guideline states that the most common possibility of resistance development among new beta-lactams under treatment is again CAZ-AVI [7]. Global and regional resistance data report CAZ-AVI susceptibility rates as high as 100% in MBL-negative Enterobacteriaceae isolates [8], [9]. Among the studies investigating the molecular mechanisms of CAZ-AVI resistance in CRE isolates, bla_KPC_ gene mutations are the most commonly reported. There are also rare reports about the CAZ-AVI resistance associated with the *bla_OXA-48_* gene [10].

OXA-48 producing CRKP was first demonstrated in Turkey [11]. In later years, OXA-48 spread rapidly among *K. pneumoniae* strains via plasmids and was detected in many regions of the world, becoming the dominant type in some regions [12], [13]. Conducting surveillance studies showing clonal relationships between bacteria that are not usually included in laboratory routines is extremely important to predict and prevent outbreaks caused by these strains [14]. Due to the prevalence of OXA-48-producing isolates in Turkey, the purpose of this study was to determine CAZ-AVI susceptibility in *bla_OXA-48_*-positive CRKP isolates in order to shed light on treatment approaches, contribute to the epidemiological data of the world and our region, and provide data for studies to investigate the role of changes in the *bla_OXA-48_* gene in CAZ-AVI resistance. An examination of the CAZ-AVI susceptibility of *bla_OXA-48_*-positive CRKP isolates grown in the laboratory of our hospital in 2020 showed a resistance rate that was quite high compared to Turkish and international data. We suspected that this was a monoclonal outbreak and aimed to perform clonal analysis on CRKP isolates. 

## Materials and methods

### Selection of isolates

Fifteen *K. pneumoniae* isolates grown in the Microbiology Laboratory of Tokat Gaziosmanpasa University Hospital in 2020, were determined as *bla_OXA-48_*-positive CRKP by the VITEK 2 compact system and multiplex PCR and were included in the study. 

### Antimicrobial susceptibility testing

Susceptibility to ertapenem, imipenem, meropenem, amikacin, gentamicin, cipro/levofloxacin, and trimethoprim-sulfamethoxazole (SXT) was determined with the VITEK-2 (bioMérieux, France) automated system. The broth microdilution method was used to investigated colistin susceptibility. Ceftazidime-avibactam susceptibilities of the isolates were tested using the Kirby-Bauer disk diffusion method with a 10/4-µg ceftazidime-avibactam disk. Escherichia coli ATCC 25922 was used as the control strain. The CAZ-AVI sensitivity results were evaluated according to the European Committee on Antimicrobial Susceptibility Testing (EUCAST) v14.0 guideline.

### Molecular detection of carbapenemase genes

DNA of CRKP isolates was extracted with the QIAamp DNA midi kit (Qiagen, Hilden, Germany). Detection of of *bla_OXA-48_, bla*_NDM_*, bla_KPC_, bla_IMP_* and *bla_VIM_* genes was performed by in-house multiplex PCR according to the standard conditions and primer sequences previously described [15]. GeneAmp PCR System 9700 (Applied Biosystems, Waltham, MA, USA) thermocycler system used for DNA amplification. Gel electrophoresis was used to visualize amplification products.

### Investigation of clonal relationships between isolates

The arbitrary primer sequence-based PCR (AP-PCR) method was used in clonal analysis of the isolates. For AP-PCR, first, 50 µl of reaction mixture including M13 primer (5’-GAGGGTGGCGGTTCT-3’) was prepared. Subsequently, amplification products were obtained with the Thermal Cycler GeneAmp PCR System 9700 (Applied Biosystems, Waltham, MA, USA) under the operating conditions described previously [16]. GelCompar II software (version 6.5; Applied Maths, Sint-Martens-Latem, Belgium) was used for band profile analysis. Cluster analysis was performed with UPGMA (Unweighted pair group method with arithmetic mean). Isolates with a Dice similarity coefficient above 95% were determined as the same genotype. If this coefficient was between 85% and 95%, the isolates were defined as subtypes of the same strain. Isolates with similarity rates below 85% were evaluated as genotypically different. 

### Ethical approval

Approval of Tokat Gaziosmanpasa University Faculty of Medicine Ethics Committee (No:23-KAEK-282) was granted on 19.12.2023.

## Results

### Distrubition of samples

Of the CRKP, 80% (12/15) were isolated from the intensive care unit (ICU), and 20% (3/15) from the ward. The distribution of the isolates according to department is shown in Table 1 (Tab. 1). The majority of the isolates were taken from blood samples 40%. This was followed by urinary system samples, with 33.3%, and sputum with 26.7%.

### Carbapenemase gene and antimicrobial susceptibility testing results

One of the 15 *bla_OXA-48_*-positive CRKP isolates was also a *bla_NDM_* carrier. All 15 *bla_OXA-48_*-positive CRKP isolates were resistant to meropenem and imipenem, amikacin, gentamicin, ciprofloxacin, and levofloxacin. Four (26.7%) CRKP isolates were susceptible to SXT and colistin. 73.3% (11/15) of the *bla_OXA-48_*-positive CRKP isolates were determined to be resistant to CAZ-AVI. Three of the four isolates susceptible to CAZ-AVI, SXT and colistin were isolated from patients staying in the ward. All 11 isolates resistant to CAZ-AVI were isolated from intensive care unit patients. 

### Clonal relationship results

**Figure 1:**
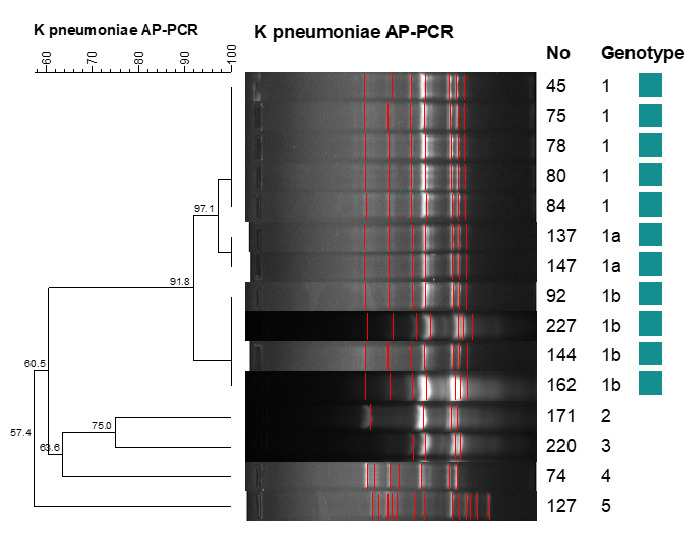
Gel electrophoresis results and dendrogram of the isolates

According to AP-PCR results, 15 isolates showed 5 different genotypes. It was determined that the clustered isolates belonged to a single distinct cluster (with a tolerance of 1.0, an optimization of 1.0, and a limit value of 85%). Eleven of the isolates were classified under genotype 1, leading to a clustering rate of 73.3%. Isolates in the genotype 1 group were defined as outbreak isolates. Figure 1 (Fig. 1) shows the gel electrophoresis images and dendrogram of the isolates.

**Figure 2:**
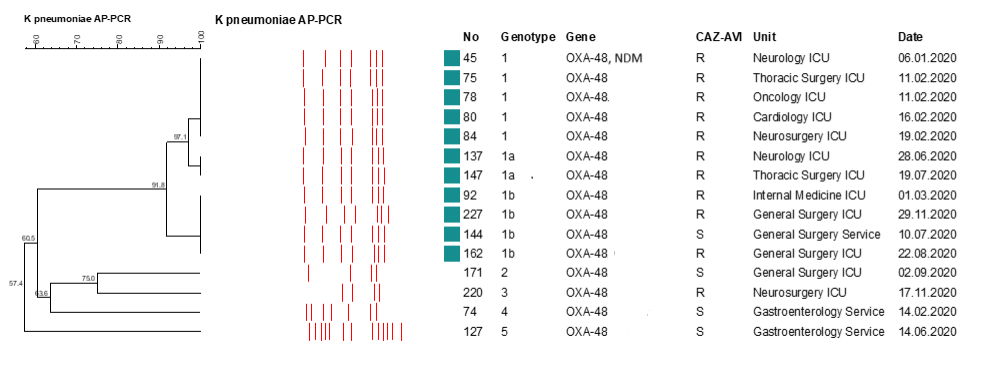
Arbitrarily primed polymerase chain reaction (AP-PCR) results of blaoxa-48-positive carbapenem-resistant K. pneumoniae isolates

One of the outbreak isolates was a *bla_OXA-48_* + *bla_NDM_* carrier. Six of the genotype 1 isolates caused an outbreak in the ICU in January through March, 2020. Four isolates were isolated in June through August, 2020. Three of these were isolated from intensive care unit patients, and one was isolated from a patient in the general surgery ward. However, this patient was transferred to the ward after staying in the general surgery ICU for 12 days. Of the four isolates that were not outbreak isolates (genotype 2, 3, 4, 5), two were isolated from ward patients and two from ICU patients (Figure 2 (Fig. 2)).

Ten (90.9%) of the outbreak isolates were resistant to all antibiotics tested, including CAZ-AVI and colistin. One of the outbreak isolates was found to be susceptible to CAZ-AVI. Three of the four isolates which were not outbreak isolates were found to be sensitive to CAZ-AVI, while one of them was resistant. The antimicrobial susceptibility profile and genotypic pattern of the isolates are shown in Table 2 (Tab. 2).

## Discussion

While almost all *Enterobactericeae* strains were sensitive to carbapenems in the early 2000s, sensitivity rates have decreased to 50% currently, varying between regions and countries [17]. Epidemiological studies show that CRKP constitutes a significant majority of CRE isolates. In the CRACKLE-2 cohort study conducted in the USA, 57% of CRE isolates were CRKP [18]. Similarly, in a study by Wang et al. in China, 1,201 of 1,801 CRE isolates were CRKP [19]. The European antimicrobial resistance surveillance report of 2023 showed that CRKP isolates in Turkey increased from 32.5% to 49.1% in 4 years [20].

Carbapenemase production in CRKP isolates is the first-line resistance mechanism in the development of carbapenem resistance. Globally, the most common carbapenemases produced in CRKP isolates are KPC, NDM and OXA-48 [17], [18], [19]. KPC-producing CRKP isolates are endemic in many countries, especially in the USA, Brazil, China and Greece [1], [17], [18], [19]. Rocha et al. detected the *bla_kpc_* gene in 94.7% of CRKP isolates in Brazil and Tsilipounidaki et al. detected the *bla_KPC_* gene in 75% of* K. pneumoniae* isolates in Greece [21], [22]. In contrast, epidemiological data in Turkey showed that OXA-48 was the most common carbapenemase in CRKP isolates. In a study within the scope of the European Survey on Carbapenemase Producing Enterobacteriaceae (EuSCAPE) project, the OXA-48 gene was detected in 83.1% of CRKP isolates in Turkey [4]. Subsequently, 6.5% NDM, 3.2% VIM, 2.4% OXA-48+NDM and 2.4% OXA-48+VIM were found. No KPC-positive isolates were detected in that study [4]. 

The rapid increase in CRE globally has created a need for new drug options in treatment. The new beta-lactam-beta-lactamase inhibitor complex ceftazidime-avibactam is used in systemic infections caused by CRE [7]. Studies investigating the *in-vitro* activity of CAZ-AVI on CRE isolates reported high susceptibility rates in MBL-negative carbapenem-resistant isolates. According to the INFORM global surveillance report, between 2015 and 2017, the susceptibility rate to caz-avi in carbapenemase-positive MBL-negative Enterobacteriaceae isolates was 99.8% [8]. In a recent study the susceptibility of ceftazidime to avibactam was reported as 84.2% in CRE isolates. In that study, 97.8% of KPC positive isolates, 69.2% of OXA-48 positive isolates, 2.6% of MBL positive isolates and 96% of carbapenemase non-producers were found to be susceptible to CAZ-AVI. It was stated that all OXA-48 positive isolates that were resistant to caz-avi were also MBL carriers [23]. In Turkey, Arici et al. [24] found an 89.3% rate of CAZ-AVI sensitivity in CRKP isolates. This increased to 100% in CRKPs that produce only OXA-48. It was determined that all CAZ-AVI resistant isolates were OXA-48+ NDM carriers [24]. In our study, 11 (73.3%) of 15 *bla_OXA-48_*-positive CRKP isolates were determined to be resistant to CAZ-AVI. Moreover, only one of them was a carrier of NDM. Our findings differ from global data and Turkey overall, the authors suspected that this was a monoclonal CAZ-AVI-resistant *bla_OXA-48_*-positive CRKP outbreak. The AP-PCR results presented here confirm this.

Hospital outbreaks of OXA-48-producing CRKP have been reported in many countries around the world. The majority of these were caused by strains isolated from patients in ICUs. The long stay of patients infected with multidrug-resistant strains in ICUs constitutes one of the predisposing factors for outbreaks caused by these resistant strains. Ten of the 11 outbreak isolates in our study were isolated from ICU patients. One isolate was from a patient transferred from the ICU to the ward. An outbreak of colistin and CRKP ST307, which produces both NDM and OXA-48, was reported in Germany in 2019 [25]. Sharma et al. [26] reported a hospital outbreak caused by 2 different sequence type clones, 56.25% of which were *bla_oxa-48_* and *bla_ndm_* producing colistin-resistant CRKP isolates. They stated that the outbreak started in the ICU. Guducuoglu et al. [27] reported a high mortality hospital outbreak in Turkey caused by colistin-resistant-, NDM- and OXA-48- positive ST11-type CRKP. These studies did not mention the CAZ-AVI susceptibility profile of the isolates. In Turkey, Arici et al. [24] reported an outbreak of OXA-48-producing CRKP originating from a single clone; all of the isolates here were susceptible to CAZ-AVI. In our study, 10 (90.9%) of the genotype 1 outbreak isolates were resistant to all antibiotics studied, including CAZ-AVI and colistin. 

Studies investigating CAZ/AVI resistance in OXA-48-producing CRKP isolates are quite limited. In a few studies, Pro68Ala and Tyr211Ser amino acid substitutions were held responsible for the decreased CAZ/AVI susceptibility in these strains. It was shown that the variant enzyme had a high ability to hydrolyze CAZ, while the inhibitory effect of AVI was reduced [28]. 

Our study is remarkable in terms of the hospital outbreak of the blaOXA-48-positive CRKP clone resistant to CAZ-AVI, which is recommended as the first choice in the treatment of CRKP infections, with reported sensitivity rates of up to 100% in OXA-48-producing CRKP. Determination of phenotypic and genotypic resistance types in hospitals is crucial for selecting treatment strategies.

In our hospital, in addition to routine infection control measures, when an increase in the frequency of an infection caused by a single microorganism with a similar antibiotic resistance profile is noticed, infection control measures are tightened. This includes increasing patient isolation measures, providing training to staff and preventing staff reassignment, and increasing the frequency of hospital environment and equipment disinfection. CAZ-AVI began to be used in Turkey on April 28, 2021. Ceftazidime-avibactam was not used in our hospital in 2020. In addition, clonal analysis and molecular resistance gene analysis are not performed routinely for bacteria. Therefore, a limitation of the present study is that active surveillance was not performed and the data were evaluated retrospectively. 

## Conclusion

Determination of phenotypic and genotypic resistance types in hospitals is important for determing appropriate treatment strategies. It is very important to conduct active surveillance studies in hospitals to prevent the spread of CAZ-AVI-resistant CRKP isolates. In addition, it is essential to routinely screen samples from ICUs, where these strains are common, and implement strict infection control programs.

## Notes

### Authors’ ORCIDs

- Taskin Dalgiç BÇ: 0000-0002-1271-7522
- Tanriverdi ES: 0000-0002-0449-0356
- Budak I: 0009-0004-3681-859X
- Yenisehirli G: 0000-0001-7030-0752
- Otlu B: 0000-0002-6220-0521

### Funding

None. 

### Competing interests

The authors declare that they have no competing interests.

## Figures and Tables

**Table 1:**
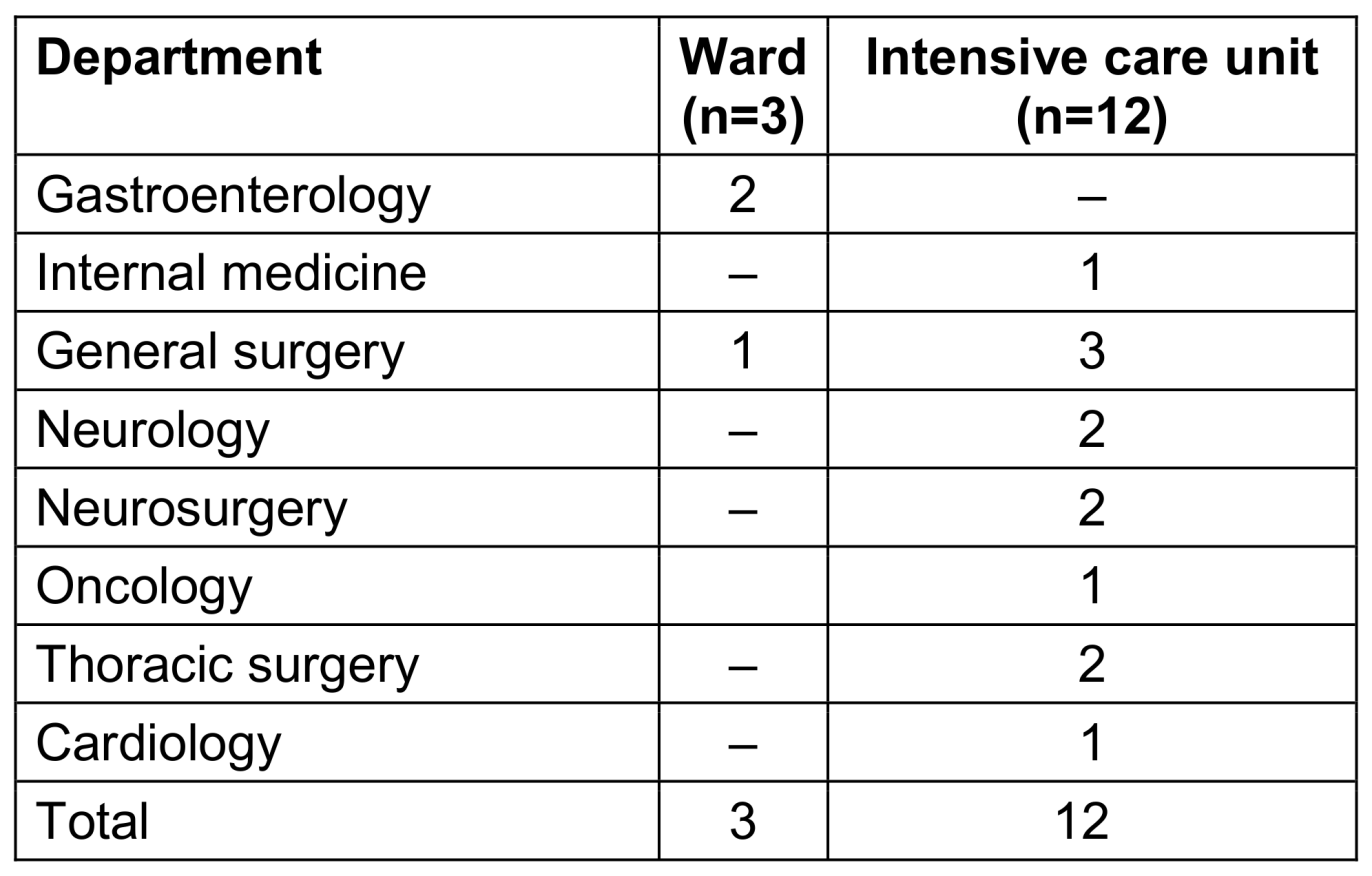
Distrubition of samples

**Table 2:**
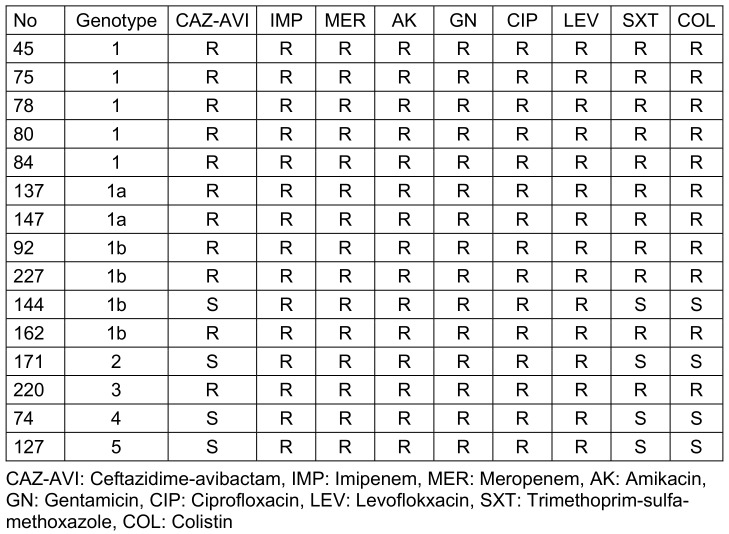
Antimicrobial susceptibility profile and genotypic pattern of the isolates
